# Supplementary material for: Multi‐dataset identification of innovative feature genes and molecular mechanisms in keratoconus
Source: J Cell Mol Med. 2024 Sep 19;28(18):e70079. doi: 10.1111/jcmm.70079 (PMC11412914; doi:10.1111/jcmm.70079)
Supplement: Supplementary file 2 — Material S2. [file JCMM-28-e70079-s002.zip › 7.GSEA analysis/1.ARL11/KEGG_ADHERENS_JUNCTION.html]

Details for gene set KEGG\_ADHERENS\_JUNCTION[GSEA]

|  || Dataset | ARL11.group.cls#h\_versus\_l |
| Phenotype | group.cls#h\_versus\_l |
| Upregulated in class | l |
| GeneSet | KEGG\_ADHERENS\_JUNCTION |
| Enrichment Score (ES) | -0.30493808 |
| Normalized Enrichment Score (NES) | -1.2088995 |
| Nominal p-value | 0.3029703 |
| FDR q-value | 1.0 |
| FWER p-Value | 0.982 |
Table: GSEA Results Summary

  

Fig 1: Enrichment plot: KEGG\_ADHERENS\_JUNCTION      
 Profile of the Running ES Score & Positions of GeneSet Members on the Rank Ordered List

  

| SYMBOL | TITLE | RANK IN GENE LIST | RANK METRIC SCORE | RUNNING ES | CORE ENRICHMENT || 1 | TGFBR2 | na | 2250 | 0.279 | -0.0150 | No |
| 2 | RAC3 | na | 4612 | 0.179 | -0.0512 | No |
| 3 | IGF1R | na | 5874 | 0.140 | -0.0626 | No |
| 4 | FARP2 | na | 6299 | 0.129 | -0.0517 | No |
| 5 | BAIAP2 | na | 6373 | 0.127 | -0.0311 | No |
| 6 | CREBBP | na | 7701 | 0.098 | -0.0519 | No |
| 7 | SMAD2 | na | 8821 | 0.078 | -0.0703 | No |
| 8 | SNAI1 | na | 9537 | 0.067 | -0.0790 | No |
| 9 | SORBS1 | na | 9757 | 0.063 | -0.0740 | No |
| 10 | INSR | na | 10038 | 0.060 | -0.0714 | No |
| 11 | SMAD4 | na | 10504 | 0.054 | -0.0752 | No |
| 12 | TCF7L2 | na | 10815 | 0.050 | -0.0753 | No |
| 13 | CTNND1 | na | 11094 | 0.047 | -0.0750 | No |
| 14 | EGFR | na | 11115 | 0.046 | -0.0673 | No |
| 15 | IQGAP1 | na | 11226 | 0.045 | -0.0624 | No |
| 16 | ACP1 | na | 12227 | 0.034 | -0.0852 | No |
| 17 | CSNK2A1 | na | 12274 | 0.034 | -0.0805 | No |
| 18 | ACTB | na | 12961 | 0.027 | -0.0954 | No |
| 19 | ACTN4 | na | 13347 | 0.024 | -0.1022 | No |
| 20 | ACTN1 | na | 13743 | 0.021 | -0.1099 | No |
| 21 | SMAD3 | na | 14170 | 0.017 | -0.1191 | No |
| 22 | CTNNB1 | na | 14653 | 0.014 | -0.1305 | No |
| 23 | CTNNA1 | na | 15057 | 0.011 | -0.1402 | No |
| 24 | NLK | na | 15607 | 0.007 | -0.1548 | No |
| 25 | TJP1 | na | 15790 | 0.005 | -0.1592 | No |
| 26 | ERBB2 | na | 16056 | 0.003 | -0.1662 | No |
| 27 | RAC1 | na | 16390 | 0.001 | -0.1756 | No |
| 28 | CDH1 | na | 16747 | -0.002 | -0.1856 | No |
| 29 | WAS | na | 17023 | -0.003 | -0.1929 | No |
| 30 | PTPRF | na | 17091 | -0.004 | -0.1942 | No |
| 31 | CSNK2B | na | 18138 | -0.011 | -0.2224 | No |
| 32 | TCF7 | na | 18185 | -0.012 | -0.2216 | No |
| 33 | WASL | na | 18335 | -0.013 | -0.2237 | No |
| 34 | CDC42 | na | 18361 | -0.013 | -0.2221 | No |
| 35 | PTPRJ | na | 18439 | -0.013 | -0.2220 | No |
| 36 | RAC2 | na | 18868 | -0.016 | -0.2314 | No |
| 37 | ACTN2 | na | 19694 | -0.022 | -0.2514 | No |
| 38 | TGFBR1 | na | 19919 | -0.023 | -0.2537 | No |
| 39 | CSNK2A2 | na | 20099 | -0.024 | -0.2545 | No |
| 40 | ACTG1 | na | 20219 | -0.025 | -0.2534 | No |
| 41 | VCL | na | 20263 | -0.026 | -0.2501 | No |
| 42 | PTPN1 | na | 20274 | -0.026 | -0.2457 | No |
| 43 | WASF1 | na | 20406 | -0.027 | -0.2447 | No |
| 44 | PARD3 | na | 21300 | -0.034 | -0.2645 | No |
| 45 | RHOA | na | 21886 | -0.038 | -0.2745 | No |
| 46 | MAP3K7 | na | 22108 | -0.041 | -0.2736 | No |
| 47 | MAPK3 | na | 22614 | -0.046 | -0.2799 | No |
| 48 | FER | na | 23484 | -0.056 | -0.2950 | Yes |
| 49 | PTPN6 | na | 23706 | -0.058 | -0.2909 | Yes |
| 50 | EP300 | na | 23862 | -0.060 | -0.2846 | Yes |
| 51 | SRC | na | 24048 | -0.063 | -0.2787 | Yes |
| 52 | TCF7L1 | na | 24530 | -0.070 | -0.2801 | Yes |
| 53 | WASF2 | na | 24555 | -0.070 | -0.2683 | Yes |
| 54 | MAPK1 | na | 24626 | -0.071 | -0.2576 | Yes |
| 55 | YES1 | na | 24999 | -0.076 | -0.2548 | Yes |
| 56 | CTNNA2 | na | 26542 | -0.099 | -0.2815 | Yes |
| 57 | WASF3 | na | 26550 | -0.100 | -0.2639 | Yes |
| 58 | FYN | na | 26579 | -0.100 | -0.2468 | Yes |
| 59 | SSX2IP | na | 27445 | -0.118 | -0.2508 | Yes |
| 60 | MET | na | 27578 | -0.120 | -0.2330 | Yes |
| 61 | LMO7 | na | 28976 | -0.156 | -0.2455 | Yes |
| 62 | SNAI2 | na | 29519 | -0.173 | -0.2302 | Yes |
| 63 | PTPRM | na | 30301 | -0.198 | -0.2174 | Yes |
| 64 | LEF1 | na | 32575 | -0.292 | -0.2307 | Yes |
| 65 | CTNNA3 | na | 32684 | -0.300 | -0.1803 | Yes |
| 66 | PTPRB | na | 33643 | -0.374 | -0.1412 | Yes |
| 67 | ACTN3 | na | 34217 | -0.455 | -0.0764 | Yes |
| 68 | FGFR1 | na | 34437 | -0.510 | 0.0084 | Yes |
Table: GSEA details [plain text format]

  

Fig 2: KEGG\_ADHERENS\_JUNCTION      
 Blue-Pink O' Gram in the Space of the Analyzed GeneSet

  

Fig 3: KEGG\_ADHERENS\_JUNCTION: Random ES distribution      
 Gene set null distribution of ES for **KEGG\_ADHERENS\_JUNCTION**
